# Beyond Integration: Unresolved Causal and Temporal Challenges in Multi‐Domain QT Risk Modeling

**DOI:** 10.1002/clc.70394

**Published:** 2026-06-23

**Authors:** Muhammad Mudasir, Shafaq Rafiq

**Affiliations:** ^1^ Jinnah Sindh Medical University Karachi Pakistan

Dear Editor,

We read with interest the framework recently proposed by Farjam et al. [1], which brings together molecular mechanisms, multi‐factorial risk factors, and AI‐based tools for predicting QT prolongation and sudden cardiac death. The authors are to be commended for integrating genetic, metabolic, pharmacological, and computational domains within a single conceptual model. We would, however, like to draw attention to two methodological issues that, in our view, merit further consideration before this framework can be translated into clinical practice: its reliance on associative rather than causal relationships, and its lack of explicit temporal modeling across data streams operating on very different timescales.

First, the framework primarily reflects associative relationships rather than causal ones. In a condition such as QT prolongation, where multiple interacting pathways converge on a single phenotype, correlation alone can obscure the true drivers of risk. An observed association between metabolic dysfunction and QT prolongation, for instance, does not establish that one causes the other, and without an explicit causal framework, this distinction remains unresolved. This matters most at the point of clinical translation: a clinician acting on a risk model needs to know not only what is associated with risk, but what can actually be modified to reduce it. Embedding causal structure into predictive models—an approach increasingly emphasized in the causal‐inference and explainable‐AI literature—would substantially improve both the interpretability and the clinical actionability of the proposed framework [2, 3].

A second, related limitation is the framework's implicit treatment of heterogeneous data streams as though they were temporally commensurate. In practice, the variables it integrates operate on markedly different timescales: genetic predisposition is fixed over a lifetime, metabolic derangements evolve over months to years, pharmacological effects fluctuate over hours to days, and electrophysiological parameters such as the QT interval can change from 1 min to the next. Combining these inputs without an explicit temporal architecture risks producing unstable or misleading risk estimates, since the clinical relevance of any given variable depends heavily on its timing. A transient, drug‐induced QT prolongation, for example, could be weighted equivalently to a chronic metabolic abnormality if temporal context is not built into the model. Incorporating time‐aware and longitudinal modeling approaches—an active area of development in healthcare machine learning—would help the framework capture dynamic risk trajectories more reliably and reduce bias arising from misaligned data sources [4, 5].

In conclusion, Farjam et al. [1] have offered a valuable conceptual synthesis for multi‐domain QT risk modeling, but its translation into clinical practice remains premature without addressing these two issues. Incorporating causal‐inference methods and time‐aware, longitudinal modeling would meaningfully strengthen the framework's interpretability, clinical applicability, and predictive reliability. We encourage the authors to consider these refinements in future iterations of the framework, so that AI‐assisted cardiac risk‐stratification tools can be deployed safely and equitably in real‐world clinical settings.

## Author Contributions

Muhammad Mudasir conducted the critique and identified the limitation points. Shafaq Rafiq wrote the introduction and conclusion.

## Funding

The authors have nothing to report.

## Ethics Statement

The authors have nothing to report.

## Conflicts of Interest

The authors declare no conflicts of interest.

## Declaration of AI Use

AI (Claude Sonnet 4.6) was used to improve the flow of the manuscript and help with minor language problems.

## Data Availability

The authors have nothing to report.
